# The elimination of HIV/AIDS depends on prevention and treatment strategies that address health inequalities

**DOI:** 10.1590/0102-311XEN106825

**Published:** 2025-07-21

**Authors:** Laio Magno

**Affiliations:** 1 Departamento de Ciências da Vida, Universidade do Estado da Bahia, Salvador, Brasil.; 2 Instituto Gonçalo Muniz, Fundação Oswaldo Cruz, Salvador, Brasil.; 3 Instituto de Saúde Coletiva, Universidade Federal da Bahia, Salvador, Brasil.

Four decades after the onset of the HIV/AIDS pandemic, we are experiencing a key moment of technological advancement capable of transforming the global epidemiological scenario, despite the many challenges ahead ^1^. We could move towards the elimination of HIV/AIDS as a public health problem. This means that we would reduce transmission of the virus to such low levels that the disease would no longer constitute a significant threat to collective health. The paths adopted to achieve elimination include reducing new infections to minimum levels through high coverage of antiretroviral therapy (ART) with viral suppression and the assurance of equitable access to prevention measures ^2^.

Recent optimism in the scientific community and among international agencies regarding the elimination of HIV as a public health problem is supported by notable advances. Several studies around the world ^3^ ^,^ ^4^ ^,^ ^5^ with hundreds of serodifferent couples (i.e., couples in which one person is living with HIV and the other is not) have shown that individuals with an undetectable viral load do not transmit the virus sexually (known as “undetectable = untransmittable”). This scientific evidence was used to establish the Joint United Nations Programme on HIV/AIDS (UNAIDS) 95-95-95 goals for the testing coverage, treatment coverage, and viral suppression. This goal for 2030 consists of diagnosing 95% of people living with HIV, treating 95% of these people with ART, and achieving 95% viral suppression of those undergoing treatment.

According to data from UNAIDS ^6^, 91% of people living with HIV in Brazil were aware of their serology in 2024, 81% of these people were undergoing ART, and 95% were in viral suppression. The two important gaps are found in access to the diagnosis and the linking of people living with HIV to health services and, consequently, access to ART.

The diversity of testing strategies, with the provision of conventional, rapid testing, and the distribution of self-tests, has been identified as effective at reaching populations in situations of greater vulnerability ^7^ ^,^ ^8^. The first gap in the Brazilian goal draws attention to the need for strengthening rapid testing programs at primary care units, especially in the context of prenatal care, but also the importance of expanding the HIV testing strategy beyond the walls of healthcare units to reach the most vulnerable populations. Although Brazil has had successful experiences with testing beyond the walls, such as the Live Better Knowing program and the distribution of self-tests in the Brazilian Unified National Health System (SUS, acronym in Portuguese), the first goal indicates the need to expand these strategies. Monitoring the self-testing strategy in Brazil shows that this strategy is still mainly accessed by white and Asian individuals (51%), gay men, other men who have sex with men (MSM), and individuals 30 to 49 years of age ^9^.

The biggest gap in the goal is the linking of people living with HIV to ART. The current scenario poses considerable risk to the health of such individuals, as it implies an increase in mortality. People living with HIV not linked to ART are likely part of populations in a situation of greater vulnerability. One study ^10^ that investigated the intersectional effects of social markers of the difference on the incidence, mortality, and lethality of AIDS in a retrospective cohort of 28.3 million individuals in Brazil, between 2007 and 2015, showed that black individuals, with less education, and those with a lower income had the poorest indicators: higher incidence, mortality, and lethality rates. Moreover, an additive interaction was found among these markers, indicating that the combined effects exceed the sum of the individual risks ^10^. These findings show how social-structural inequalities intensify the vulnerability to illness and death from AIDS, highlighting race/skin color as a critical marker of these inequalities and the urgent need for public policies directed at equity in education and income for historically marginalized populations.

Prophylactic strategies using antiretrovirals constitute another available measure, such as post-exposure prophylaxis (PEP) and pre-exposure prophylaxis (PrEP) in both oral and recent long-acting injectable formulations. These prevention methods have demonstrated high efficacy in preventing HIV if used properly and with high adherence. Oral PrEP has been reported to be one of the main factors for the reduction in new cases of HIV throughout the world, such as in Scotland ^11^, the city of Montreal in Canada ^12^, and the state of New South Wales in Australia ^13^. This technology has been available in the SUS since 2018 and was likely one of the factors that helped reduce the number of cases of HIV by 53% between 2016 and 2024 in the city of São Paulo ^14^.

Although injectable PrEP is not yet widely available, studies using mathematical models predict a significant impact on reducing the occurrence of new HIV cases when coverage is expanded ^15^ ^,^ ^16^ ^,^ ^17^. However, such models have the limitation of not adequately taking into account the negative impact of social inequities in different countries, which may lead to an overestimation of the positive impact of PrEP. The price of injectable formulations is also a significant barrier to access in countries with limited economic resources. In the international political and economic realm, negotiations with the global pharmaceutical industry continue to be a crucial obstacle to expanding the availability of novel injectable formulations in the Global South. High costs and resistance to patent flexibility on the part of large pharmaceutical companies constitute challenges that need to be addressed through advocacy and more assertive public policies.

Although it is necessary to control the pandemic, the passive provision of these technologies in the SUS is not sufficient to ensure the elimination of HIV. PrEP has often been viewed only as a biomedical technology for combined prevention, as described in the manuals ^18^. This perspective may underestimate the importance of the social and political dimensions involved in its implementation. Several studies state that adherence to and the continuation of PrEP do not depend exclusively on individual behavior, but are influenced by broader social, economic, and cultural contexts ^19^ ^,^ ^20^. Stigma, racism, sexism, homophobia, and transphobia constitute significant barriers that impede equal access to prevention, even in countries with universal healthcare systems, such as Brazil ^21^.

Although the Brazilian Ministry of Health, in collaboration with researchers and civil society, has invested efforts to expand access to PrEP (e.g., the implementation of dispensing machines, expansion of prescriptions in primary care, and encouragement of telehealth), recent data indicate that significant portions of the population remain unassisted, especially those in situations of greater social vulnerability. For example, among the population using PrEP in Brazil up to May 2025, 55% self-identified as white or Asian, 71% reported having 12 or more years of schooling, 81% self-identified as cis gay men or MSM, and 42% were between 30 and 39 years of age ^22^. These data show that we are still failing to reach black and brown individuals, trans individuals, cis women, and individuals in situations of socioeconomic vulnerability; in other words, we have excluded the populations most in need of the current scientific advances.

We also need to consider the rise of extremist movements in several Western countries, which poses new threats to the human rights agenda, directly impacting the fight against HIV/AIDS ^21^. Budget cuts promoted by governments such as the United States have compromised international prevention and care programs, negatively impacting regions that have historically been exploited and depend on these resources, especially on the African continent. A simulation study ^23^ showed that the suspension of international funding through the U.S. President’s Emergency Plan for AIDS Relief (PEPFAR), determined by an executive order of the United States in January 2025, showed that 60,000 additional deaths from HIV are projected by 2030 in the most conservative scenario. Even for scenarios in which the programs were resumed after four or eight weeks, 21,000 to 28,000 additional deaths would be expected, in addition to an increase of 35,000 to 103,000 new cases of HIV infection ^23^.

The availability of PrEP in Brazil has made significant progress but still faces structural and bureaucratic obstacles. To address these obstacles, the Brazilian Ministry of Health issued a document entitled *Guidelines for the Elimination of AIDS and HIV Transmission as Public Health Problems in Brazil by 2030* ^24^. In this document, PrEP is addressed as an essential component of combined prevention. Priority actions include decentralization and the availability of PrEP in municipalities distant from major cities, with a focus on expanding access in regions and populations with low coverage, especially adolescents, young people, gay men, MSM, trans individuals, and sex workers. The document also proposes specific strategies for promoting adherence to the use of PrEP, such as peer support and the use of digital technologies, as well as the implementation of long-acting formulations. Communication and social mobilization are emphasized, with educational campaigns aimed at expanding knowledge and the acceptance of PrEP among priority populations ^24^.

The effective implementation of PrEP, the expansion of testing, and ART linkage strategies require not only technological and scientific advances, but also a deep understanding of the social and political dimensions that affect access and adherence. The challenges are multiple and include aspects that range from structural and bureaucratic issues to international obstacles related to the pharmaceutical industry and emerging conservative policies. It is therefore essential to strengthen decentralization and community mobilization strategies, in addition to promoting inclusive public policies that ensure equitable access to prevention for the most vulnerable populations.

The current context requires a broader, more coordinated mobilization of civil society, activists, researchers, and public administrators to push for bolder, more inclusive policies. It is necessary to revive the spirit of previous initiatives that ensured universal HIV treatment in Brazil, including the courageous policy of patent flexibilization. The further decentralization of the provision of PrEP is fundamental, with the inclusion of this strategy in the routine of community services, increasing prescriptions by non-medical professionals (such as nurses, pharmacists, etc.), and promoting strategies that reduce the local micro-powers that hinder universal access. Ensuring that PrEP is truly available to all who need it is more than a technical issue; it is a fundamental ethical and political issue for contemporary Brazil.

It is essential to address persistent barriers to the diagnosis and treatment of individuals living with HIV by expanding access to testing, including actions beyond the walls of healthcare units, the use of self-tests, and the prioritization of populations in situations of high vulnerability. These processes must be accompanied by effective mechanisms for linking patients to care and antiretroviral therapy. Public policies are needed that integrate testing actions with community strategies for patient reception and social support, ensuring that all individuals diagnosed with HIV initiate and maintain treatment in a timely manner to achieve sustained viral suppression. Confronting the pandemic, therefore, requires an integrated approach that goes beyond the provision of biomedical technologies and addresses health inequities.
