# Supplementary material for: The elimination of HIV/AIDS depends on prevention and treatment strategies that address health inequalities
Source: Cad Saude Publica. 2025 Jul 21;41(7):e00106825. doi: 10.1590/0102-311XEN106825 (PMC12337695; doi:10.1590/0102-311XEN106825)
Supplement: Translation

# Translation: A eliminação do HIV/aids depende de estratégias de prevenção e tratamento que enfrentem as iniquidades em saúde

**DOI:** 10.1590/0102-311XPT106825

**Authors:** Laio Magno

**Affiliations:** 1 Departamento de Ciências da Vida, Universidade do Estado da Bahia, Salvador, Brasil.; 2 Instituto Gonçalo Muniz, Fundação Oswaldo Cruz, Salvador, Brasil.; 3 Instituto de Saúde Coletiva, Universidade Federal da Bahia, Salvador, Brasil.

Após quatro décadas da pandemia de HIV/aids, vivemos um momento-chave de avanço tecnológico capaz de transformar o cenário epidemiológico mundial, embora haja ainda muitos desafios pela frente ^1^. Há a possibilidade de caminharmos em direção da sua eliminação como problema de saúde pública. Isso significa que reduziríamos a transmissão do vírus a níveis tão baixos que isso deixaria de representar uma ameaça significativa à saúde coletiva. Entre os caminhos adotados para alcançar a eliminação estão a redução das novas infecções a níveis mínimos através da alta cobertura de terapia antirretroviral (TARV) com o alcance de supressão viral e a garantia de equidade no acesso às medidas de prevenção ^2^ .

O recente otimismo na comunidade científica e entre agências internacionais em relação à eliminação do HIV como problema de saúde pública é sustentado por avanços notáveis. Diversos estudos no mundo ^3^ ^,^ ^4^ ^,^ ^5^, com centenas de casais sorodiferentes (i.e., os casais em que uma pessoa vive com HIV e a outra não), mostraram que indivíduos com carga viral indetectável não transmitem o vírus por via sexual (conhecido como “indetectável = intransmissível”). Essas evidências científicas foram usadas para o estabelecimento das metas 95-95-95 do Programa Conjunto das Nações Unidas sobre HIV/AIDS (UNAIDS, acrônimo em inglês) para cobertura de testagem, tratamento e supressão viral. Essa meta para 2030 consiste em diagnosticar 95% das pessoas vivendo com HIV, tratar 95% dessas pessoas com TARV e alcançar a supressão viral em 95% das que estão em tratamento.

Segundo dados da UNAIDS, o Brasil apresentou, em 2024, 91% das pessoas vivendo com HIV cientes da sorologia, 81% dessas pessoas em TARV e 95% dessas pessoas em supressão viral ^6^. As duas lacunas importantes estão localizadas no acesso ao diagnóstico e na vinculação das pessoas vivendo com HIV aos serviços de saúde e, consequentemente, o acesso à TARV.

A diversificação das estratégias de testagem, com a oferta de testagem convencional, rápida e distribuição de autotestes, tem sido apontada como efetiva para alcançar populações em situação de maior vulnerabilidade ^7^ ^,^ ^8^. A primeira lacuna da meta brasileira chama atenção para necessidade de fortalecimento de programas de testagem rápida em unidades da atenção primária à saúde, especialmente no contexto do pré-natal, mas também para a importância da expansão de estratégia de testagem de HIV que ultrapasse os muros das unidades de saúde e que alcance as populações mais vulnerabilizadas. Embora o Brasil tenha tido experiências exitosas com a testagem extramuros, como o programa Viva Melhor Sabendo e distribuição de autotestes no Sistema Único de Saúde (SUS), a primeira meta indica a necessidade de avanço dessas estratégias. O monitoramento da estratégia de autotestes no Brasil mostra que essa estratégia ainda é majoritariamente acessada por pessoas brancas e amarelas (51%), homens gays e outros homens que fazem sexo com homens (HSH) e com idade de 30 a 49 anos ^9^.

A maior lacuna da meta é a vinculação das pessoas vivendo com HIV à TARV. Esse cenário representa um grande risco à saúde dessas pessoas, pois implica em aumento da mortalidade. Provavelmente, as pessoas que vivem com HIV que não estão sendo vinculadas fazem parte de populações em situação de maior vulnerabilidade. Estudo que avaliou os efeitos interseccionais dos marcadores sociais da diferença sobre a incidência, mortalidade e letalidade por aids, em uma coorte retrospectiva de 28,3 milhões de indivíduos no Brasil, entre 2007 e 2015, mostrou que pessoas negras, com menor escolaridade e menor renda apresentaram os piores indicadores: maior taxa de incidência, de mortalidade e de letalidade. Além disso, observou-se interação aditiva positiva entre esses marcadores, indicando que os efeitos combinados superam a soma dos riscos individuais ^10^. Esses achados evidenciam como desigualdades socioestruturais amplificam a vulnerabilidade ao adoecimento e à morte por aids, ressaltando a raça/cor como marcador crítico dessas desigualdades e a urgência de políticas públicas voltadas à equidade em educação e renda para populações historicamente marginalizadas.

Outra tecnologia disponível são as estratégias profiláticas com o uso de antirretrovirais, como a profilaxia pós-exposição (PEP) e a profilaxia pré-exposição (PrEP), tanto oral quanto nas recentes formulações injetáveis de longa duração. Esses métodos de prevenção já demonstraram alta eficácia na prevenção do HIV, se forem usados de forma adequada e com alta adesão. A PrEP oral foi relatada em alguns estudos como um dos principais fatores para redução de novos casos de HIV no mundo: na Escócia ^11^, na cidade de Montreal no Canadá ^12^ e no Estado de Nova Gales do Sul na Austrália ^13^. Esse método está disponível no SUS desde 2018 e, provavelmente, foi um dos fatores que contribuíram para a redução de 53% dos casos de HIV entre 2016 e 2024 na cidade de São Paulo ^14^.

Embora a PrEP injetável ainda não tenha sido amplamente disponibilizada no mundo, estudos com modelos matemáticos projetam grande impacto na redução de novos casos de HIV quando houver a ampliação da cobertura dessa tecnologia ^15^ ^,^ ^16^ ^,^ ^17^. Esses modelos, entretanto, possuem a limitação de não levarem em conta adequadamente o impacto negativo das iniquidades sociais existentes nos diversos países, o que pode superestimar o impacto positivo da PrEP. Destaca-se, ainda, o preço dessas tecnologias injetáveis como uma barreira importante para o acesso de países com poucos recursos econômicos. No plano político e econômico internacional, as negociações com a indústria farmacêutica global continuam sendo um obstáculo crucial para ampliar a disponibilidade das novas formulações injetáveis no Sul Global. Altos custos e resistência à flexibilização de patentes por parte das grandes empresas farmacêuticas são desafios que precisam ser enfrentados por meio de *advocacy* e políticas públicas mais incisivas.

Embora seja necessária para o controle da pandemia, a disponibilização passiva dessas tecnologias no SUS não é suficiente para garantir a eliminação do HIV. A PrEP frequentemente tem sido encarada apenas como uma tecnologia biomédica de prevenção combinada, conforme descrito nos manuais ^18^. Essa perspectiva pode subestimar o peso das dimensões sociais e políticas envolvidas em sua implementação. Diversos estudos destacam que a adesão e continuidade da PrEP não dependem exclusivamente do comportamento individual, mas são influenciadas por contextos sociais, econômicos e culturais mais amplos ^19^ ^,^ ^20^. Fenômenos como estigma, racismo, sexismo, homofobia e transfobia constituem barreiras significativas que impedem o acesso igualitário à prevenção, mesmo em países com sistemas universais de saúde, como o Brasil ^21^.

Embora o Ministério da Saúde brasileiro, em colaboração com pesquisadores e sociedade civil, tenha investido esforços para ampliar o acesso à PrEP (e.g., a implementação de máquinas dispensadoras, expansão da prescrição na atenção primária, e o incentivo à telessaúde), dados recentes indicam que parcelas significativas da população, especialmente aquelas em situação de maior vulnerabilidade social, permanecem desassistidas. Por exemplo, da população que está em uso de PrEP no Brasil, até maio de 2025, 55% se autodeclarou branco ou amarelo, 71% declarou possuir 12 anos ou mais de estudo, 81% se autodeclarou homem cis gay ou HSH e 42% está na faixa etária de 30 a 39 anos ^22^. Esses dados mostram que ainda não estamos conseguindo alcançar pessoas negras (i.e., pretos e pardos), pessoas trans, mulheres cis, pessoas em situação de vulnerabilidade socioeconômica, ou seja, temos excluído essas populações mais necessitadas dos avanços científicos atuais.

Nesse contexto, também precisamos destacar que o avanço de movimentos extremistas em vários países ocidentais traz novas ameaças à agenda dos direitos humanos, refletindo-se diretamente no enfrentamento ao HIV/aids ^21^. Cortes orçamentários promovidos por governos como os Estados Unidos, por exemplo, têm comprometido programas internacionais de prevenção e assistência, impactando negativamente regiões historicamente exploradas e dependentes desses recursos, especialmente no continente africano. Estudo de simulação ^23^ mostrou que a suspensão do financiamento internacional por meio do Plano de Emergência do Presidente dos Estados Unidos para o Combate à Aids (PEPFAR, acrônimo em inglês), determinada por ordem executiva dos Estados Unidos em janeiro de 2025, mostrou que no cenário mais conservador projetaram-se 60 mil mortes adicionais por HIV até 2030. Esse estudo também mostrou que mesmo em cenários em que os programas fossem retomados após 4 ou 8 semanas, seriam esperadas de 21 mil a 28 mil mortes adicionais, além de um aumento nas novas infecções por HIV que varia entre 35 mil e 103 mil casos ^23^.

A disponibilização de PrEP no Brasil tem tido um avanço significativo, mas enfrenta ainda entraves estruturais e burocráticos. Para o enfrentamento desses entraves, o Ministério da Saúde lançou o documento *Diretrizes para a Eliminação da Aids e da Transmissão do HIV como Problemas de Saúde Pública no Brasil até 2030* ^24^. Nesse documento, a PrEP é abordada como componente essencial da prevenção combinada. Entre as ações prioritárias estão a descentralização e interiorização da PrEP, com foco na ampliação do acesso em regiões e populações com baixa cobertura, especialmente adolescentes, jovens, gays e outros HSH, pessoas trans e profissionais do sexo. Além disso, o documento propõe estratégias específicas para promover a adesão e retenção ao uso da PrEP, como o apoio de pares e o uso de tecnologias digitais, bem como a implementação das formulações de longa duração. A comunicação e mobilização social também são enfatizadas, com campanhas educativas voltadas à ampliação do conhecimento e aceitação da PrEP entre as populações prioritárias ^24^.

A implementação eficaz da PrEP, a ampliação de estratégias de testagem e a vinculação à TARV requerem não apenas avanços tecnológicos e científicos, mas também uma profunda compreensão das dimensões sociais e políticas que envolvem a adesão e o acesso. Os desafios são múltiplos e incluem desde questões estruturais e burocráticas até obstáculos internacionais relacionados à indústria farmacêutica e políticas conservadoras emergentes. Assim, é fundamental fortalecer estratégias de descentralização e mobilização comunitária, além de promover políticas públicas inclusivas que garantam acesso equitativo à prevenção para as populações mais vulneráveis.

O contexto atual exige uma mobilização ampliada e articulada da sociedade civil, ativistas, pesquisadores e gestores públicos para pressionar por políticas mais ousadas e inclusivas. É necessário retomar o espírito das iniciativas anteriores que garantiram o tratamento universal do HIV no Brasil, incluindo a corajosa política de flexibilização de patentes. É fundamental descentralizar ainda mais a oferta de PrEP, incluindo-a na rotina de serviços comunitários, ampliando prescrições por profissionais não médicos (como enfermeiros, farmacêuticos e outros), e promovendo estratégias que reduzam os micropoderes locais que dificultam o acesso universal. Garantir que a PrEP esteja realmente ao alcance de todos que dela necessitam é mais do que uma questão técnica; é uma questão ética e política fundamental para o Brasil contemporâneo.

Além disso, torna-se indispensável enfrentar de forma contundente as barreiras persistentes no diagnóstico e tratamento das pessoas vivendo com HIV através da ampliação do acesso à testagem, incluindo as estratégias extramuros, uso de autotestes e priorização de populações em contexto de alta vulnerabilidade. Esses processos devem ser acompanhados por mecanismos eficazes de vinculação ao cuidado e à terapia antirretroviral. Faz-se necessário investir em políticas públicas que integrem ações de testagem com estratégias comunitárias de acolhimento, navegação de pacientes e apoio social, assegurando que todas as pessoas diagnosticadas com HIV iniciem e mantenham o tratamento em tempo oportuno, com vistas à supressão viral sustentada. O enfrentamento à pandemia exige, portanto, uma abordagem integrada, que vá além da disponibilização de tecnologias biomédicas e enfrente as iniquidades em saúde.
